# Poor-Law Infirmaries as Training Schools

**Published:** 1913-07-19

**Authors:** G. J. Masson Martin

**Affiliations:** Senior Assistant Medical Officer Portsmouth Infirmary.


### POOR-LAW INFIRMARIES AS TRAINING SCHOOLS.

By G. J. MASSON MARTIN, L.R.C.P. & S. I., D.P.H., Senior Assistant Medical Officer Portsmouth Infirmary.

### I.—For The Newly Qualified.

Of recent years the whole outlook of the medical profession has been changed. This change is noticeable not only from the standpoint of medical men themselves but also from that of the lay public. The old conception of a doctor as a grey-whiskered, spectacled, benevolent individual in well-worn frock-coat and tall hat, armed with an inevitable black bag, which to the infant mind shared equal honours with a stork as a representative of the source of generation, is fast dying out. While we will hope that the kindliness and undoubted "healing-power" of this older school is retained by the rising generation of practitioners, the coming of asepsis and modern exact scientific methods of diagnosis and treatment render the representation of a smarter, more alert type to be that required by modern society.

In olden days the three years required by the medical curriculum of that time was sufficient in which to carry out the course of study which then represented the science of medicine. Later on it was found necessary to increase this period by two years, and at present the five years laid down by the General Medical Council for the course is hardly sufficient in which to cram all the advances which the recent researches in all branches of medicine have added to the original groundwork. The student will be fortunate if he is able to pass all his examinations successfully in these five years and at the end of that time he will find that it will be necessary to spend another year at least for the assimilation of the knowledge he has already obtained and the acquirement of the power of putting it into practice, if he wishes to be thoroughly efficient and capable of responding to all the calls which the profession now makes on her devotees.

For those who are fortunate enough to be able to afford the time and money necessary if they wish to become consultants in any branch of their profession, the attainment of an appointment in the large teaching hospital in which they received their medical education naturally presents itself as the best, and, indeed, almost the necessary sequela to their qualification; but for the great majority, who look forward to general practice as their ultimate means of livelihood, and who wish to take the immediate opportunity of relieving the strain on their own or their parents' resources which the possession of a diploma affords, this is neither necessary nor advisable.

It is here that the large Poor-Law infirmary presents itself as a suitable institution in which the newly qualified medical man can perfect himself in the carrying out of his work, before launching out into the open and uncharted sea of private practice. It is only the larger infirmaries, however, which are useful in this respect—that is, those which are under the administration of a medical superintendent and employ at least two assistant medical officers, and it must be understood that instruction in such institutions only is that treated of in this article. Although larger salaries may be obtained in smaller institutions, such appointments are not suitable for newly qualified men.

House appointments in large teaching hospitals are usually honorary, the *cachet* which such an appointment gives being considered sufficient recompense for the services rendered, while the junior resident appointments in infirmaries, although not over well paid, afford means of living in comparative comfort on the salary they provide.

For many reasons Poor-Law infirmaries are equal if not superior to other general medical institutions, such as provincial hospitals, in teaching the carrying out of methods of treatment, and in enabling that self-confidence to be learnt which the practitioner will find of so much value to him in his later work. In almost all provincial hospitals the cases are divided amongst a number of honorary visiting physicians and surgeons under whose charge they are placed and for the treatment of whom they are responsible. The house surgeon acts merely as an assistant to this honorary staff, and generally has no cases which are directly under his care and control. The diagnosis and treatment of all the patients are made and ordered by the visiting doctors, and he can only see that such treatment is adequately carried out, with the opportunity of treating minor symptoms himself as they arise.

In a large Poor-Law infirmary the position held by a resident is entirely different. For one thing the number of cases in such institutions far exceeds those in most charitable hospitals, and besides this the resident has a certain number of cases directly under his care, diagnosing and prescribing treatment for a case, looking after it all through, and being responsible for its welfare from beginning to end. He, of course, is under the control and guidance of the medical superintendent for the carrying out of such treatment, but as a rule the care of a certain number of cases is under his entire control, the medical superintendent acting only as a consultant in exceptional cases which present some unusual difficulty. It will be easily understood that having this almost complete control of a great number of cases of all sorts of disease will be of much more service in cultivating an accurate sense of diagnosis and of self-reliance in treatment than when patients are only nominally under him, and when the responsibility rests mainly on the shoulders of a visiting honorary staff.

The great number of cases in such an institution will also present to the resident opportunities for carrying out minor surgical operations such as will be useful to him later in practice; he will also have the opportunity of seeing the exact state of affairs revealed at major operations at which he will assist, in cases in which he is responsible for the first diagnosis as well as for the after treatment. He may also be allowed to take the opportunity of testing new methods of treatment. Some mention was made of tuberculin treatment at infirmaries in a former number of this periodical. An extensive trial of a remedy of this nature could not be carried out by a resident in an institution such as a provincial hospital, where the various divergent views of the many members of the honorary staff would make wholesale treatment of this sort impossible.

A great deal will, of course, depend on the medical colleagues under whom the junior resident will have to work, and this in some degree constitutes a disadvantage which such posts may present. It will be found generally, however, if those who have control of the newly appointed resident find that his work is carried out thoroughly and conscientiously, that facilities for special treatment will not be withheld.

Another advantage which most Poor-Law infirmaries hold over the greater number of general hospitals in granting experience which will be of use later on in private practice is in the great mainstay of such practice—namely, midwifery. In almost all infirmaries a lying-in ward or maternity block is provided and the resident medical men are called in to attend any cases of difficult labour which may arise. The normal cases are, as a rule, conducted by a midwife. A knowledge of the treatment of mental disease may also be obtained by practice in the imbecile blocks which are generally attached to workhouse infirmaries, and a separate department for diseases of the eye is also sometimes provided. Experience may be obtained, too, in the treatment of venereal disease, as Lock wards are usually a feature in such institutions. In short, the class of cases which present themselves for treatment in workhouse infirmaries are exactly the same as those which a practitioner meets in his daily practice, and a resident in such an institution will have the advantage that the treatment of such cases will be carried out and learnt under almost ideal conditions, and any mistakes which may be made —and no experience of any worth is gained without the commission of *some* mistakes—can be more easily discovered and remedied without injury to the patient or loss of prestige on the part of the physician than if they were met with in private practice.

The advantage of gaining such experience is obvious, and it will be found that a year or so spent in a large Poor-Law infirmary by a newly qualified medical man will not be time wasted, and may be very useful to him during his subsequent career.

